# The Story of the Insane from Year to Year

**Published:** 1901-06-01

**Authors:** 


### THE STORY OF THE INSANE FROM YEAR TO YEAR.

(Thirteenth Series.)

### BETHLEHEM ROYAL HOSPITAL.

On January 1st, 1899, this venerable institution contained 225 patients, and at the end of December the number had fallen to 188. There were 126 first admissions, 53 readmissions, 91 recoveries, 31 discharged relieved, 76 not improved, and 18 died. The average number resident was 217, and the total number under treatment was 400. Calculated on the admissions the recoveries stand at 50 per cent., and calculated on the average number resident the deaths stand at 8·3 per cent. Compared with the County Asylum averages the percentage of recoveries is very high and the percentage of deaths rather low, but these differences are accounted for by the fact that as a rule only recent and presumably curable cases are admitted. The assigned cause of insanity in 63 of the year's admissions was such moral causes as domestic trouble, mental anxiety and overwork, and among the physical causes, intemperance in drink accounts for 12 and hereditary influence for 72. Of the patients admitted during 1899 not fewer than 58 were sent in under urgency orders, 27 were voluntary boarders, and 34 were paying cases. This is one of the Lunatic Hospitals in which much clinical instruction is given. Dr. Hyslop teaches a class from St. Mary's Hospital, Dr. Craig one in connection with the London Post Graduate Course, and Drs. Savage, Rayner, and Percy Smith have given clinical instruction to students from Guy's, St. Thomas's, and Charing Cross. In addition to all this a limited number of students attended the practice of the hospital for periods of three months.

